# Single-molecule approaches for DNA damage detection and repair: A focus on Repair Assisted Damage Detection (RADD)^☆^

**DOI:** 10.1016/j.dnarep.2023.103533

**Published:** 2023-09

**Authors:** Tahir Detinis Zur, Jasline Deek, Yuval Ebenstein

**Affiliations:** School of Chemistry, Center for Nanoscience and Nanotechnology, Center for Light-Matter Interaction, Raymond and Beverly Sackler Faculty of Exact Sciences, Tel Aviv University, Tel Aviv, Israel

**Keywords:** DNA damage, DNA repair, Single-molecule, Repair Assisted Damage Detection (RADD), Fluorescence microscopy, Single-strand DNA damage, Damage quantification, Repair enzymes, Repair dynamics, Optical genomic mapping

## Abstract

The human genome is continually exposed to various stressors, which can result in DNA damage, mutations, and diseases. Among the different types of DNA damage, single-strand lesions are commonly induced by external stressors and metabolic processes. Accurate detection and quantification of DNA damage are crucial for understanding repair mechanisms, assessing environmental impacts, and evaluating response to therapy. However, traditional techniques have limitations in sensitivity and the ability to detect multiple types of damage. In recent years, single-molecule fluorescence approaches have emerged as powerful tools for precisely localizing and quantifying DNA damage. Repair Assisted Damage Detection (RADD) is a single-molecule technique that employs specific repair enzymes to excise damaged bases and incorporates fluorescently labeled nucleotides to visualize the damage. This technique provides valuable insights into repair efficiency and sequence-specific damage. In this review, we discuss the principles and applications of RADD assays, highlighting their potential for enhancing our understanding of DNA damage and repair processes.

## Highlights

• Single-strand DNA damage is the most common yet the least explored form of DNA damage.
• Single-molecule fluorescence assays provide the ultimate sensitivity for damage detection by counting individual lesions along extended DNA molecules.
• Repair enzyme cocktails used for DNA damage detection may be tailored to label specific damage lesions or combined for broad spectrum detection.
• Tracking the number of damage sites over time provides valuable insights into cell repair dynamics.
• DNA damage may be mapped to specific genomic loci by combining RADD with optical genome mapping approaches.

## 1 Introduction

In 2015, Tomas Lindahl, Paul Modrich, and Aziz Sancar received the Nobel Prize in Chemistry for their pioneering work in the field of DNA damage and repair. Their achievements underscore the vital importance of this field, which has far-reaching implications for our understanding of human health and disease [1], [2], [3].

Various stressors, originating from both external factors and cellular processes, pose a continuous threat to the integrity and functionality of the human DNA within the genome. This constant exposure increases the risk of DNA damage, potentially leading to detrimental effects on its structure and proper function. Failure to repair this damage can lead to mutations, genomic instability, and the development of diseases [4], [5]. Single-strand lesions represent the most prevalent form of DNA damage and range from physical rupture of the strands to chemical modification of the affected base. These lesions can be induced by external radiation or toxins, as well as by normal metabolic processes that result in the production of reactive oxygen species (ROS) that oxidize DNA, or by directly triggering the formation of specific DNA structures like cyclobutane pyrimidine dimers (CPDs) and 6–4 photoproducts [6].

In normal cellular conditions, highly efficient repair enzymes address DNA damage and prevent single-strand breaks (SSBs) from progressing into double-strand breaks (DSBs). Single strand damage is usually repaired by nucleotide excision repair (NER) or base excision repair (BER) processes. These mechanisms involve the excision of damaged DNA bases, creating a gap that is subsequently filled by DNA polymerase, using the complementary strand as a template [7], [8].

Detection and quantification of DNA damage are essential for elucidating the underlying mechanisms of DNA repair, assessing the impact of environmental exposures and toxins, enabling early clinical diagnosis, and evaluating the response to therapy. Various techniques have emerged in recent years to address these needs, often relying on the use of lesion-specific antibodies or DNA integrity detection methods. SSBs and DSBs are commonly measured indirectly by unwinding of the DNA. This includes the comet assay and other electrophoresis-based techniques [9], [10], [11], [12], [13]. Alternatively, immunological labeling of damage sites using fluorescent-based detection assays such as Enzyme-Linked Immunosorbent Assay (ELISA), Dot-blot, flow cytometry, and immunohistochemistry, have also been utilized [14], [15], [16], [17], [18], [19], [20], [21]. However, these techniques have limitations, including poor sensitivity and constrains in detecting multiple types of damage [16], [22]. To overcome these limitations, the single-molecule approach has emerged as a powerful tool for sensitive detection and quantification of various types of DNA damage. This approach directly detects damaged lesions within single DNA molecules, allowing for the precise localization and quantification of damage that may be masked in other large-scale assays [23], [24], [25].

In this review, we inspect single-molecule, in vitro Repair assisted damage detection (RADD) assays that rely on enzymatic repair. RADD is a DNA damage detection technique that utilizes specific repair enzymes to remove damaged bases and incorporate fluorescently labeled nucleotides, enabling precise visualization and quantification of DNA lesions. This technique involves several steps. Firstly, specific repair enzymes, such as glycosylases or endonucleases recognize and excise damaged bases from the DNA molecule. These enzymes possess high specificity, targeting particular types of damage, including oxidized lesions or base dimers. Following base excision, the resulting gaps are filled in by DNA polymerases using fluorescently labeled nucleotides as building blocks. The incorporated fluorescent nucleotides are detectable under fluorescence microscopy, allowing for the precise localization and quantification of the damaged DNA regions.

## 2 Broad spectrum detection of multiple types of ss-DNA damage lesions

Detection of the total burden of ss-DNA damage is a crucial aspect of understanding the complex landscape of DNA damage. Various types of ss-DNA lesions can arise from different sources and have distinct implications for genome stability and cellular function. There are several commercially available repair enzyme cocktails that address a wide spectrum of DNA damage. One example is the PreCR Repair Mix® (New England Biolabs, USA). This cocktail is typically used as a pre-treatment step before PCR amplification to improve the success rate of amplification from damaged or degraded DNA samples.

**Fig. 1:**
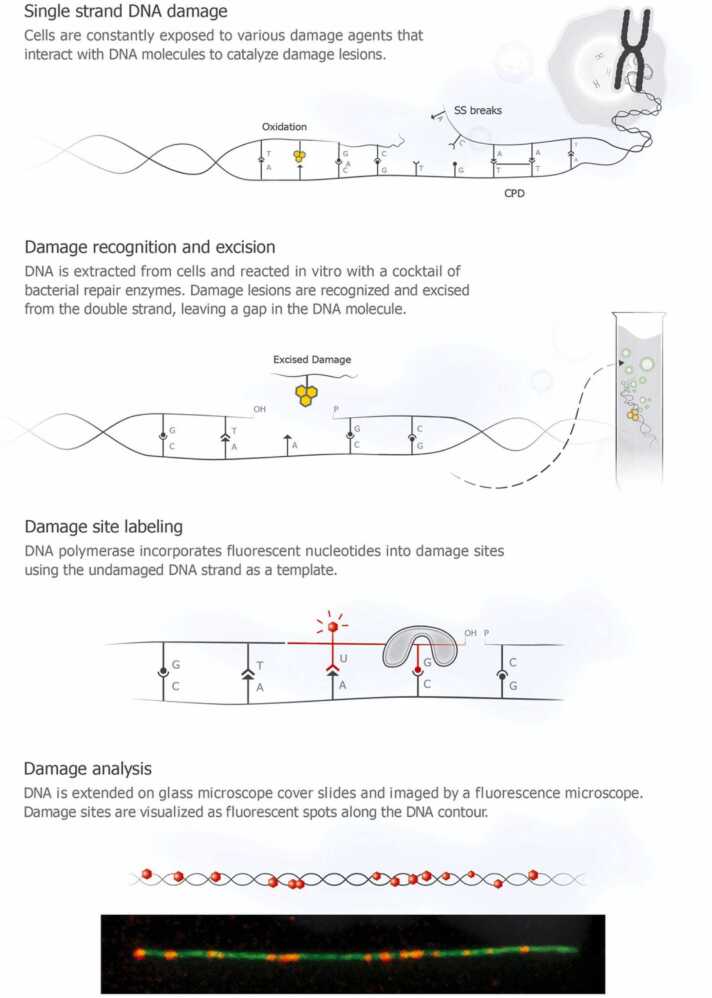
Schematic depiction of the RADD method illustrating the recognition and repair of DNA damage lesions in vitro. In this process, the damaged lesions are excised and substituted with fluorescent nucleotides by DNA polymerase. The labeled DNA is then extended on glass slides and visualized using a fluorescence microscope, allowing the repaired damage sites to be observed as fluorescent spots along the DNA molecule. Reprinted with permission from [24] Copyright 2014 American Chemical Society.

Zirkin et al. [24] utilized this mix for RADD by adding fluorescent nucleotides, thus labeling a broad spectrum of DNA lesions, such as abasic sites, uracils, oxidative damage, and ultraviolet(UV)-induced damage. The experimental procedure is illustrated in Fig. 1. Initially, DNA was extracted from U2OS cells that have been exposed to increasing intensities of UV radiation. The purified genomic DNA was then treated with a PreCR Repair Mix®, comprising bacterial DNA glycosylases and endonucleases: Endonuclease (Endo) IV, Endo VIII, formamidopyrimidine DNA glycosylase (FpG), T4 pyrimidine dimer glycosylase (PDG), uracil-DNA glycosylase (UDG), and Bst DNA polymerase. These enzymes recognized and removed the DNA damage, while the DNA polymerase incorporated fluorescently labeled nucleotides to label the sites of damage. The DNA molecules were subsequently stretched on modified glass coverslips and imaged using a fluorescence microscope. The number of damage sites was quantified by counting the spots per length of DNA molecules in Mbp (damage sites per million base pairs). The latest technical protocols were presented by Torchinsky et al. [26].

**Fig. 2:**
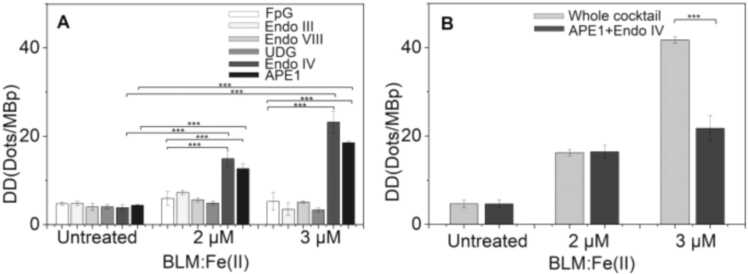
DNA damage was detected in untreated and BLM:Fe(II)-treated PBMCs from five healthy individuals. The samples were individually incubated with one repair enzyme at a time from the mentioned enzyme cocktail (A). The samples were incubated with either the complete enzyme cocktail or only APE1 and Endo IV (B). By Singh et al. used under CC BY 4.0.

In a series of studies using a modified assay, damage caused by various factors, including ionizing radiation (IR) and hyperthermia [27], Bleomycin [28], Etoposide [29], PhenDC3 [30], and novel metallodrugs [31], was quantified. Singh et al. investigated the effects of chemotherapeutic drugs Bleomycin and Etoposide as well as IR and hyperthermia on PBMCs using a repair enzyme cocktail consisting of Apurinic/apyrimidinic Endonuclease 1 (APE1), FpG, Endo III, Endo IV, Endo VIII, and UDG. APE1 and Endo IV are essential for repair of apurinic/apyrimidinic (AP) sites, and played a crucial role in detecting damage caused by Bleomycin (see Fig. 2) and IR. McStay et al. confirmed that APE1 was the most efficient enzyme in repairing damage caused by metallodrugs, as the majority of lesions generated were AP sites, while Obi et al., found that the inclusion of FpG and Endo VIII was crucial for the detection of damage caused by the G4-stabilizing compound PhenDC3.

### 2.1 Addressing specific types of DNA damage lesions

The study mentioned earlier showcased the proficiency of the repair cocktail in identifying and repairing diverse forms of DNA damage. While it successfully dealt with a broad spectrum of DNA damage lesions, it is many times desirable to focus on and study specific types of lesions for a comprehensive understanding and targeted approach. This involves customizing the repair enzyme mix by incorporating specific enzymes tailored to the desired damage type. Numerous studies have explored these modifications, offering examples of how a specialized repair enzyme mix can be developed to effectively target specific lesions of interest.

In 2013, Lee et al. [23] conducted a study focusing on the visualization of UV-induced damage on single DNA molecules by subjecting λ DNA molecules to controlled levels of UV radiation, resulting in the generation of DSBs, SSBs, and various damage lesions. The damaged DNA molecules were treated with PDG, an enzyme that specifically digests pyrimidine dimers— TT crosslinks that are commonly caused by UV radiation. By utilizing PDG, the team was able to selectively target and cleave the damaged sites. Subsequently, DNA polymerase I incorporated fluorescently labeled nucleotides into these cleaved regions, enabling the visualization of the crosslinked dimer sites.

In another noteworthy study from 2016 [25], the same group extended their research focus to the visualization of reactive oxygen species (ROS)-induced DNA damage in DNA molecules. They achieved ROS induced damage by using Fenton reaction. ROS, known for their involvement in various physiological processes, can also induce oxidative stress and damage DNA. To visualize the ROS-induced DNA damage, the researchers utilized a similar approach as in their previous study. High molecular weight DNA molecules were exposed to ferrous chloride (FeCl_2_) and hydrogen peroxide (H_2_O_2_) inducing the Fenton reaction, resulting in the formation of oxidative lesions such as 8-oxoguanine and abasic sites. By employing a repair enzyme mix consisting of Endo III, Endo VIII, and FpG, the specific lesions were selectively targeted and eliminated, resulting in the formation of AP sites. These AP sites then served as targets for the incorporation of fluorescent nucleotides, enabling visualization of the damaged regions. Similarly, in a variant of this assay, substitution of Endo III with Endo IV, enabled the identification of oxidative DNA damage induced by alcohol in the genome of Escherichia coli [32].

**Fig. 3:**
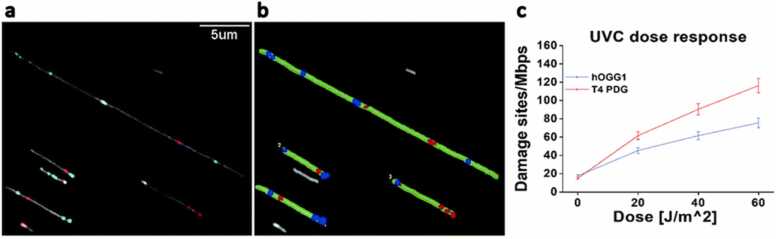
Simultaneous detection and quantification of photoproducts and oxidative DNA damage in HEK cells following exposure to UVC radiation. (a) Fluorescence image of labeled DNA molecules, with red dots indicating photoproduct sites and blue dots representing oxidative damage sites. (b) Analysis results using automatic software, with green for detected DNA molecules, red dots for photoproduct labels, and blue dots for oxidative damage sites. (c) Dose response of HEK cells exposed to UVC radiation, with red representing photoproduct levels and blue indicating oxidative damage levels. Reproduced from [26] with permission from the Royal Society of Chemistry.

**Fig. 4:**
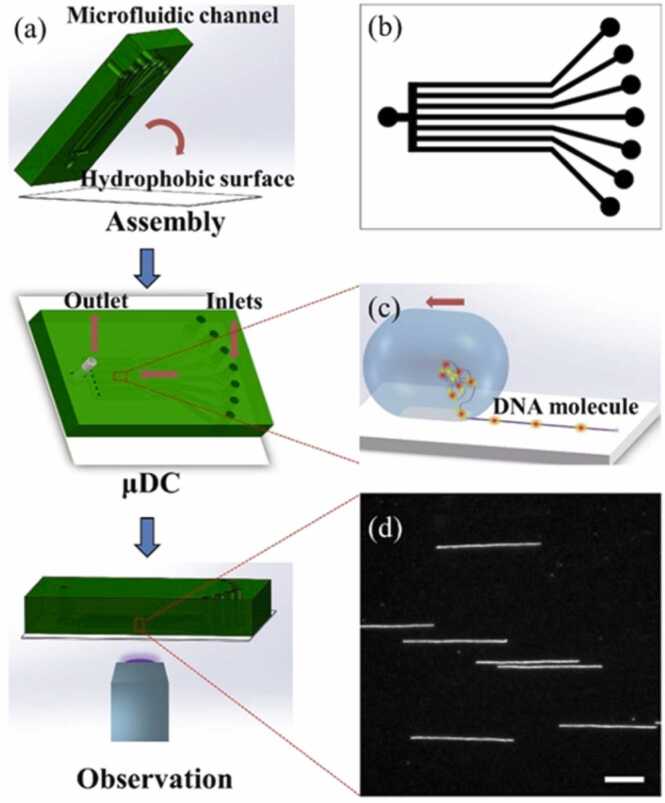
Schematic diagram illustrating microfluidic device (μDC) and fluorescence imaging process. It includes the fabrication of the device, stretching and imaging of a DNA sample, and shows stretched DNA molecules on a hydrophobic surface captured by fluorescence microscopy. From [33] © IOP Publishing. Reproduced with permission. All rights reserved.

Exposures such as UVB irradiation can damage DNA directly and indirectly, via ROS induced DNA damage. Understanding the interplay between related damage processes is essential for elucidating mechanistic pathways for damage and repair. Torchinsky et al. [26] reported a study introducing a novel assay aimed to enable single-molecule multi-color detection and relative quantification of both photoproducts and oxidative damage. To distinguish between damage types using distinct colors, the researchers employed consecutive reactions for labelling photoproducts and oxidative damage separately. As a model, HEK cells were exposed to UVA, UVB, or UVC radiation for increasing durations to induce photoproducts and related oxidative damage. The labelling of photoproducts was achieved using T4 PDG, and oxidative damage was addressed by human 8-oxoguanine DNA glycosylase (hOGG1), an enzyme that releases damaged purines from double-stranded DNA. The collected data was then analyzed using a custom, publicly available software (https://github.com/ebensteinLab/Tiff16_Analyzer) (Fig. 3b). The software accurately identified and measured the length of stretched DNA molecules, and calculated the number of labels present along the DNA contour (Fig. 3a). In order to allow higher throughput and multiplexing, a microfluidic DNA deposition device was developed [33]. UV damage measurements were performed on multiple samples by the use of a simple Polydimethylsiloxane (PDMS) stamp containing a manifold of parallel microchannels that allow detecting several samples on the same imaging substrate (Fig. 4).

### 2.2 Applying single-molecule RADD to study repair dynamics

Maintaining the integrity of the genome and preventing genetic abnormalities and instability, which are linked to conditions such as cancer, heavily rely on the essential process of DNA repair. Cells employ diverse repair pathways to detect and eliminate various forms of DNA damage, maintaining the proper function of the genome [5], [7], [8], [34]. Analyzing repair dynamics may provide a deeper understanding of the influence of various factors on repair efficiency and the interplay between repair processes and other cellular events. By performing RADD at different time points after induction of a DNA damaging agent, the rate of repair may be evaluated quantitatively, reporting on the state of cellular repair machinery.

**Fig. 5:**
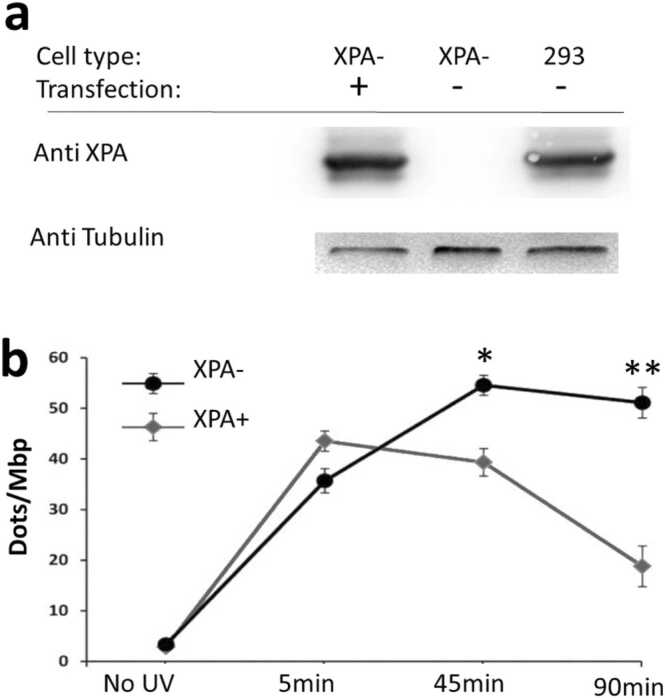
Examination of DNA repair in XP-derived cells. (a) Western blot comparing the XPA protein level in XPA-deficient cells (XPA-) with XPA-restored cells (XPA+) and HEK-293 cell line as a control. (b) Comparison of DNA repair in XPA- and XPA+ cells after UV radiation exposure, with the number of damage signals per Mbp of DNA plotted over time. Reprinted with permission from [24] Copyright 2014 American Chemical Society.

Zirkin et al. [24] utilized RADD to investigate DNA repair dynamics in various experimental settings. First, they studied the rate of UV-induced DNA damage repair in normal cells and cells overexpressing the PIM-2 protein. The results showed that PIM-2 overexpression led to more efficient repair, as evidenced by a rapid decline in the number of damage sites over time. They compared their single-molecule approach to a commercial ELISA kit and found that their method had higher sensitivity and lower noise. Furthermore, they examined the repair process in a Xeroderma pigmentosum (XP) patient derived cell line, lacking the XPA protein, which is crucial for repair of UV induced DNA damage. The XPA deficient cells exhibited impaired repair after UV irradiation, while the cells with recombinant XPA showed significantly recovered repair (Fig. 5).

Torchinsky et al. [26] tested the effect of UV irradiation on the human skin model cell line, HaCat. They tracked the repair process by allowing HaCat cells to recover for specific periods of time after UV irradiation and then measured the retention of photoproducts and oxidative lesions. Control cells without exposure to UV served as a baseline for comparison. The results demonstrated repair dynamics of HaCat cells following exposure to 1800 J/m^2^ of "environmental UV," showing an initial surge in damage levels immediately after irradiation followed by a gradual decrease over time due to natural repair mechanisms.

Using a similar methodology, another research study sought to examine the repair kinetics of DNA damage induced by IR with and without hyperthermia treatment [27]. In this study, the researchers exposed peripheral blood mononuclear cells (PBMCs) from three healthy donors to IR and incubated them for varying periods at different temperatures. While the repair rates were comparable across the samples, there was a notable difference in the quantity of remaining damage after 90 min. Notably, when hyperthermia treatment preceded IR exposure, there was an approximate twofold increase in the amount of single-stranded DNA damage, and no evidence of repair was observed after 30 min. This lack of repair post-IR may be attributed to the inhibitory effects of hyperthermia on DNA repair processes.

### 2.3 Towards genomic mapping

The field of single-molecule optical genome mapping (OGM) has witnessed significant advancements in recent years, particularly in the ability to accurately determine the genetic identity of observed DNA molecules through fluorescent barcoding. This technique provides information on genome organization, large-scale rearrangements, and cellular heterogeneity [35], [36], [37]. It is particularly useful for studying epigenetic marks and modifications, enabling the investigation of DNA methylation patterns, histone modifications, and other epigenetic features on the single molecule level [38], [39], [40], [41]. OGM, when combined with the RADD approach, holds the potential to reveal the sequence-specific locations of DNA damage sites and the variation between cells. By establishing correlations between DNA damage and the underlying sequence, along with other observable factors, a more comprehensive understanding of genomic alterations can be achieved.

The early studies in this field primarily concentrated on mapping damage lesions within the 50kbp genome of the λ bacteriophage by generating sequence-specific maps that were aligned with the damaged DNA molecules [23], [25]. Lee et al. examined the sequence dependence of UV-induced DNA damage by comparing thymine dimer (TT) frequency maps *in silico* with SSB labeled DNA molecules. The comparison suggested that the DNA sequences present in crucial genes responsible for the capsid and tail may possess a natural resistance to UV radiation, independent of DNA repair enzymes.

In a subsequent study, the same group employed restriction enzymes to divide the DNA molecules into two fragments, enabling the determination of the molecule direction. They then induced the Fenton reaction and compared *in silico* sequence frequency maps with ROS-induced DNA damage. The results revealed a correlation between specific sequences (GTGG and GTGA) and oxidative damage. Interestingly, a lower frequency of damage and GTGR sequences was found in the early left operon region, active during the lysogenic cycle. This suggests that the DNA sequence itself may influence sensitivity to oxidative damage.

**Fig. 6:**
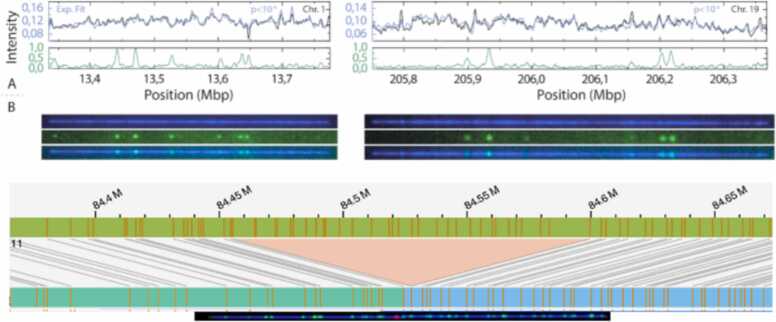
DNA damage visualization and alignment to the human genome. Upper panel: (A) DNA molecules from PBMCs exposed to etoposide. Experimental (blue) and theoretical (black) barcodes show excellent overlap (top). Normalized intensity in the 647 channel reveals DNA damage sites (green) along the DNA molecule's center line (bottom). By Muller et al. used under CC BY 4.0. (B) Microscopy images: YOYO-1 channel (blue, top), ATTO-647 channel (green, center), and composite overlay (bottom). By Muller et al. used under CC BY 4.0. Lower panel: DNA molecule from U2OS cells exposed to KBrO3 aligned to a *de novo* assembly reference map. Damage site visualized in red and barcode sites visualized in green. The reference map, which includes the DLG2 gene, reveals a significant deletion within the gene, represented by an orange triangle.

The human genome, which is orders of magnitude longer and more complex, requires separate labeling schemes in order to map DNA molecules to the genome reference, and observe damage events in their genetic context. Muller et al. [42] induced damage in PBMCs using a cytotoxic drug. The researchers performed DNA mapping using a competitive binding approach, incorporating two molecules: Netropsin and YOYO-1. By blocking the binding of YOYO-1 to AT-rich regions, Netropsin caused AT-rich regions to appear darker in comparison to GC-rich regions, creating a locus specific fluorescence intensity profile along DNA molecules extended in nanochannels. Damage sites were labeled as previously mentioned [24] and mapped to the human genome using the competitive binding maps (Fig. 6, upper panel).

In a recent work, our group conducted an in-depth analysis of DNA damage distribution at the genomic level. By utilizing the RADD method and combining it with a high-throughput nanochannel-based OGM from Bionano Genomics, we investigated oxidative damage in the genome of the U2OS osteosarcoma cell line treated with potassium bromate (KBrO3). The workflow involved extracting high molecular weight DNA, fluorescently labeling 8oxodG and sequence-specific motifs in chromosomal DNA molecules and confining the labeled DNA in silicon nanochannel arrays. The confined DNA molecules were then automatically imaged on a Saphyr instrument (BionanoGenomics Inc.), enabling simultaneous detection of genetic information and damage localization. The fluorescent marker pattern along the stretched molecules served as a unique barcode, indicating its genomic origin. These barcodes were digitized and aligned to a reference map or assembled to create consensus contiguous maps.

Through the utilization of OGM, we were able to detect the DNA damage distribution along the genome and to successfully identify a comprehensive spectrum of large structural and copy number variants (SVs and CNVs, accordingly). In the lower panel of Fig. 6, we provide an illustrative example of a DNA molecule aligned to a reference map containing the DLG2 gene, revealing a notable deletion spanning 144.224 Kbp. This finding is consistent with a recent study that identifies DLG2 as a tumor suppressor in osteosarcoma, with approximately 42 % of human osteosarcoma samples displaying DLG2 deletions [43]. Notably, a damage site appears in red in the middle of the molecule displaying the ability to map damage sites to complex genomes.

## 3 Conclusions

The RADD assay is a general concept for tagging DNA damage sites by a combination of repair enzymes and functionalized nucleotides. RADD offers several advantages over traditional techniques, including high sensitivity, the ability to detect multiple types of damage, and the capacity to study repair dynamics within cells. RADD has been successfully utilized for characterizing DNA damage in fixed cells and tissue sections [44], [45], [46], in global genomic DNA samples at high-throughput [47], and in DNA sequencing for genomic mapping [48]. Nevertheless, single-molecule detection as summarized in this report holds the highest sensitivity, with access to basal DNA damage levels and the potential of multiplexing with other epigenetic information. Furthermore, the combination of RADD with optical DNA mapping techniques holds great promise for comprehensive genomic mapping. By establishing correlations between DNA damage sites and the underlying DNA sequence at the single-molecule level, researchers may identify hotspots related to damage or repair events, correlate the with other epigenetic observables, and gain a deeper understanding of the processes underlying DNA damage and repair.

RADD is simple to perform, and the availability of protocols, analytical software, and commercial reagents may ease its adoption by the research community. This review presents examples for the utility of single-molecule RADD, showcasing its potential for further discoveries in the field of DNA damage research. It may have promising applications in enhancing our understanding of the impact of environmental exposures, pharmaceutical toxicity studies, and evaluating therapeutic interventions. Continued advancements in RADD technology and its integration with other analytical approaches will undoubtedly pave the way for exciting future developments in the field.

## Declaration of Competing Interest

The authors declare no conflicts of interest.

## Data Availability

No data was used for the research described in the article.
